# Effect of Ketoprofen on acute phase protein concentrations in goats undergoing castration

**DOI:** 10.1186/s12917-016-0748-y

**Published:** 2016-06-23

**Authors:** Umit Karademir, Ibrahim Akin, Hasan Erdogan, Kerem Ural, Gamze Sevri Ekren Asici

**Affiliations:** Department of Pharmacology and Toxicology, Faculty of Veterinary Medicine, University of Adnan Menderes, Isikli, Aydin Turkey; Department of Surgery, Faculty of Veterinary Medicine, University of Adnan Menderes, Isikli Koyu, Aydin Turkey; Department of Internal Medicine, Faculty of Veterinary Medicine, University of Adnan Menderes, Isikli Koyu, Aydin Turkey; Department of Biochemistry, Faculty of Veterinary Medicine, University of Adnan Menderes, Isikli Koyu, Aydin Turkey

**Keywords:** Acute Phase Protein, Castration, Goat, Ketoprofen

## Abstract

**Background:**

The objective of this study was to determine the effect of ketoprofen on acute phase protein (APPs) concentrations in goats undergoing castration. A total of 16 clinically healthy, male and 12 months old goats were enrolled and each case received ketoprofen (group I) or control (group II) in a randomized fashion. Goats were sedated with Xylazine-HCl, afterwards ketoprofen (3 mg/kg) was injected via jugular vein in group I, whereas physiological saline solution was administered to group II. Goats were castrated by the Burdizzo method. Hematological parameters were determined with a blood cell counter and plasma fibrinogen (Fb), serum haptoglobin (Hp), serum amyloid A (SAA) and ceruloplasmin (Cp) concentrations were measured Millars technique, ELISA kit or *p*-phenylenediamine oxidase activity prior to castration and throughout the study on 0 to 96 h.

**Results:**

There were no differences in pre-treatment serum Cp, SAA and Fb concentrations among the groups. Contrarily, there were significant differences in plasma Hp concentrations on 0 to 96 h onwards post-castration. There were no differences in WBC and PCV between groups. Cp, Fb, and SAA were almost constant or showed slight changes at various stages of the study with no significant differences between groups.

**Conclusions:**

The results revealed that, levels of Cp, Fb and SAA may not be affected by castration such as the confounding parameters similarly to stress. More investigations possessing different surgical or non-surgical castration techniques with larger number of goats and focusing on specific markers for stress are suggested for precise analysis.

## Background

Goats are one of the most important food-producing animal species in developing countries. The castration of male goats is a routine practice in many countries aimed at reducing management problems with aggressive and sexual behaviour, as well as improving meat quality [28]. The main techniques used to castrate goats include surgical or nonsurgical/ischemic (elastrator, burdizzo or emasculatome) methods [6, 8]. Castration has been shown to elicit inflammatory reactions, physiological stress, suppression of immune function, pain-associated behaviour, and a reduction in performance [15, 16, 28].

The acute phase response (APR) refers to nonspecific and complex reaction of an animal that includes changes in concentration of numerous liver derived plasma proteins, called acute phase proteins (APPs) [20]. APPs are a group of blood proteins that change in concentration in animals subjected to external or internal challenges, such as infection, inflammation, surgical trauma or stress and they are classified as positive (major, moderate and minor) or negative depending on the increase or decrease in the serum concentration, respectively, during the APR [3]. Positive APPs, such as haptoglobin, C-reactive protein, serum amyloid A, ceruloplasmin, fibrinogen, and alpha 1-acid glycoprotein, increase in concentration in response to inflammation. The “negative” APPs decrease in concentration in response to inflammation and include proteins like albumin and transferrin [30]. Quantification of APP concentration in plasma or serum can provide valuable diagnostic information in the detection, prognosis, and monitoring of disease in several animal species [12]. In addition, the use of APPs for screening in ante- or post-mortem inspection to identify animals that should be subjected to a more thorough inspection or to ensure the health of animals prior to entry to the human food chain has been suggested [38].

Nonsteroidal anti-inflammatory drugs (NSAID) including ketoprofen (KTP) are among the most widely drugs in veterinary medicine. They block the activity of cyclooxygenase (COX) enzymes and reduce prostaglandin concentrations through the body. As a consequence, inflammation, pain and fever are reduced [17, 27]. These drugs make them ideal for the clinical management of inflammation and postoperative pain in animals. However, patients condition (eg, respiratory, renal or hepatic insufficiency, dehydration, ascites, coagulopathies, pregnancy or gastric ulcer) and drugs selection must be considered prior to NSAID use due to their potential of adverse effects (eg, antithrombotic activity, gastro-duodenal erosion and ulceration, nephropathy, delayed healing or nonunion of the wound and fracture) [2, 5, 27]. KTP, a propionic acid derivate, is a NSAID which used for the treatment and management symptoms associated with musculoskeletal inflammation and pain in animals [5]. Ruminants have been investigated on the effects of APPs in experimental inflammation models and stress by many authors which the administration of a different dose of lipopolysaccharide [43], turpentine [20], some *Pasteurella spp.* (e.g., *P. haemolytica, P. multocida*) [7, 25] and virus spp. (e.g. respiratory syncytial, viral diarrhoea) [18, 22], vaccination [11] or restricted feeding [26] and transportation [29]. Although the effects of NSAIDs on APPs in castrated ruminants have been well recognized in some of the prior research articles [9, 40, 41], the effects of NSAIDs on castration induced increases in APPs in goats have not been yet reported and limited data is a currently available. Furthermore there is scarce information in goats on APPs, clearly indicating that there is a need to evaluate goat model. Hence it was hypothesized that ketoprofen has probably effects on some of the positive APPs (Hp, SAA, Fb and Cp) in relation to castration, which is a frequent procedure of goats with Ketoprofen administration.

However, to the best of our knowledge, there is no published data for alterations of APPs in castration of goats with NSAID administration. The aim of this study was to investigate the alterations of some positive APPs (Hp, SAA, Fb and Cp) in castration of goats with KTP administration.

## Results

**Fig. 1:**
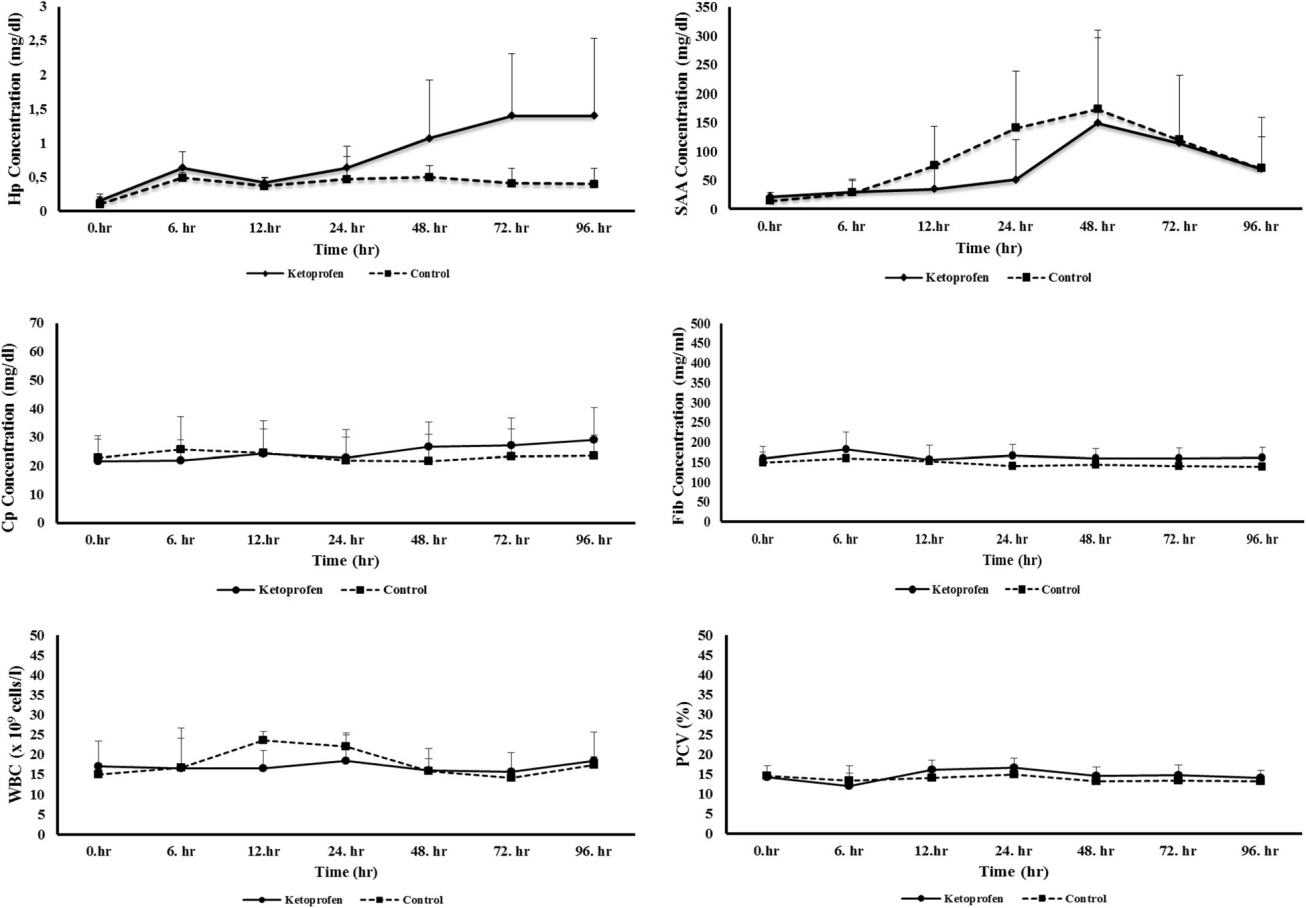
Mean ± SD concentrations of Hp, SAA, Cp and Fb as well as WBC and PCV counts in male goats (*n* = 8 goats/group) receiving iv administered control treatment and ketoprofen (3 mg/kg) before and 96 h after undergoing castration

All goats remained healthy through the study. Through available evidence suggested that castration lead to an increase in APPs in group II. Changes in mean values for serum concentrations of Hp, SAA and Cp, plasma concentrations of Fib, WBC and PCV counts were determined over the sample collection period for the two groups (Table 1). There were no differences in pre-treatment serum Cp, SAA and Fib concentrations among the groups (Fig. 1).

**Table 1:** Mean ± SD concentrations of Hp, SAA, Cp and Fb as well as WBC and PCV counts undergoing castration in male goats (*n* = 8 goats/group)

	Group	0.hr	6. hr	12.hr	24. hr	48. hr	72. hr	96. hr	Interactions	*p* value
Hp (mg/dl)	Ketoprofen	0,15 ± 0,10	0,63 ± 0,24	0,41 ± 0,09	0,63 ± 0,33	1,06 ± 0,86	1,39 ± 0,92	1,39 ± 1,15	Group	0,020
	Control	0,10 ± 0,11	0,49 ± 0,08	0,37 ± 0,12	0,47 ± 0,33	0,50 ± 0,17	0,41 ± 0,22	0,40 ± 0,23	Time	0,020
									Group by Time	0,212
SAA (mg/dl)	Ketoprofen	21,10 ± 8,01	30,05 ± 18,86	34,55 ± 38,34	51,07 ± 69,60	149,18 ± 147,04	114,43 ± 117,21	69,46 ± 89,74	Group	0,392
	Control	14,52 ± 4,42	29,37 ± 22,90	75,82 ± 67,67	140,48 ± 97,44	173,33 ± 136,34	120,72 ± 110,72	71,14 ± 54,55	Time	0,021
									Group by Time	0,433
Cp (mg/dl)	Ketoprofen	21,60 ± 7,88	21,86 ± 7,17	24,23 ± 8,62	22,78 ± 7,34	26,63 ± 8,67	27,23 ± 9,55	29,12 ± 11,16	Group	0,753
	Control	22,93 ± 7,58	25,66 ± 11,52	24,65 ± 11,27	21,92 ± 10,82	21,75 ± 9,19	23,29 ± 9,73	23,52 ± 7,22	Time	0,012
									Group by Time	0,001
Fib (mg/dl)	Ketoprofen	159,75 ± 29,64	181,78 ± 44,50	156,78 ± 35,78	165,96 ± 28,20	159,70 ± 25,10	160,29 ± 25,48	161,56 ± 25,34	Group	0,096
	Control	149,03 ± 26,18	160,58 ± 6,97	153,38 ± 7,51	139,93 ± 3,83	143,68 ± 7,59	140,48 ± 3,56	139,00 ± 6,14	Time	0,038
									Group by Time	0,003
WBC (×10^9^ cells/l)	Ketoprofen	17,04 ± 6,47	16,60 ± 7,49	16,58 ± 4,44	18,53 ± 6,50	16,08 ± 5,52	15,67 ± 4,77	18,46 ± 7,25	Group	0,571
	Control	14,96 ± 1,20	16,74 ± 9,99	23,64 ± 2,15	22,14 ± 3,35	15,91 ± 2,99	14,07 ± 2,35	17,35 ± 2,09	Time	0,000
									Group by Time	0,000
PCV (%)	Ketoprofen	14,20 ± 2,99	12,01 ± 3,19	16,15 ± 2,40	16,52 ± 2,53	14,54 ± 2,18	14,72 ± 2,53	14,09 ± 1,75	Group	0,112
	Control	14,48 ± 0,58	13,29 ± 3,89	14,00 ± 0,97	14,96 ± 1,39	13,22 ± 1,50	13,36 ± 1,54	13,18 ± 0,67	Time	0,036
									Group by Time	0,630

At the beginning of the study, there was no significant difference regarding Hp on 24 h of study. Afterwards through 48 to 96 h of the completion of the study there was a statistically significant difference between group I and II regarding Hp. The present authors also reported that there was significant group by time interaction regarding Cp (*P*= 0,001), Fb (*P*= 0,003) and WBC (*P*= 0,001) values. For other parameters (Hp, SAA and PCV) there was no group by time interaction.

Regarding SAA there was no significance between group I and II throughout the study whereas there was a time interaction in group I among 0.hr and 48 to 96 h. Besides this difference exists at 24^th^ hour in group II. Taking into account Cp values there was a time interaction through 0. and 48 to 96 h in group I. Fibrinogen values possessed significant alterations on 0 and 6^th^ hours. WBC values presented time interaction and group by time interaction (*P*<0,001).

## Discussion

Regarding veterinary literature in farm animals APPs are important diagnostic indicators of inflammatory disorders also in goats [1, 4, 10]. In the analytical methods of prior study for measuring Hp, SAA, acid soluble glycoprotein (ASG), Fb, and albumin concentrations in goats were validated, in an attempt to assess their response to an inflammatory stimulus in goats [20]. In a recent article establishing reference intervals for acute phase proteins in healthy goats; Hp was be interpreted with caution in unknown pregnancy status, besides it was also suggested that APPs were recommended as useful biomarkers in goat diseases [4]. Hp increases were reported in several diseases of goats; i.e. helminth infestations [42], ruminal acidosis [19], sarcoptic mange [36], besnoidosis [31], coccidiosis [21] and gangrenous mastitis [13]. On the other hand there was no statistically significant differences were found regarding Hp concentrations in Caprine arthritis encephalitis positive and negative goats [24].

The present authors interest to this subject was aroused following receipt of goats referred for castration. At that time a through literature search revealed studies in cattle subjected to castration in relation to APPs. Contrarily the present authors were unaware of finding documented reports regarding APPs and castration procedure in goats.

As aforementioned above cattle studies largely took a part in the literature. In a prior trial determining the effect of repeated KTP administration to surgically castrated bulls on APPs revealed increased plasma Hp and Fb concentrations were increased (*P*<0.05) on day 3 in the castration groups in comparison to the controls, in which were attributed to tissue trauma induced by castration. In the latter study surgical castration increased plasma cortisol and acute-phase proteins. On the other hand repeated KTP dose 24 h after treatment did not have influence on alteration in APPs [41]. Similarly, the effects of carprofen administration before banding or burdizzo castration of bulls on APPs were investigated. In that study Hp concentrations presented similarity (*P*= 0.58) among treatments before the time of castration. Afterwards on day 1, no differences in Hp concentration was detected and castrated and control groups. On day 3 band group showed elevated (*P*<0.05) Hp in comparison to control. On the other hand no differences in Hp concentrations were detected among treatments on d 7, 14, 21, and 28. finally on day 35 banded group showed greater (*P*<0.05) Hp concentrations compared with Band+C and control groups [34].

In the present study, Hp was the solely affected APPs deemed statistically significant in KTP administered goats (*P*<0.05) in comparison to controls. This may be briefly explained. Although recognized of the concentrations of the Hp, Cp and Fb may be useful in the diagnosis of tissue injury [35], according to the results of the present study unlike Hp, levels of Cp, Fb, and SAA may not be affected by tissue injury through Burdizzo castration. Increased Hp levels observed in this study might be related to the immediate tissue trauma, inflammation, and probably psychological (pain) stress in response to castration [32, 34]. Pang et al. [33] reported banding or burdizzo castration did not effect plasma Hp and Fb levels. Previous reports presented an increase in Hp and Fb levels on days 1, 3, and 7 post-castration in younger animals [14, 15, 34, 40]. Horadagoda et al. [23] reported that APPs, such as SAA and Hp are excellent markers for indicating acute inflammatory conditions in cattle. Pang et al. [33] stated that unchanged Hp and Fb levels in castrates, might be related to the dynamics (increased followed by a return to normal) of APPs during injury. In addition WBC and PCV values were deemed statistically unaffected between groups, revealed that tissue damage or injury was not significant, nor stress leukogram appeared in castrated animals participated in the present study.

## Conclusion

Cp, Fb and SAA were almost constant or showed slight changes at various stages of the study with no significant difference between groups. Levels of Cp, Fb and SAA is not affected by castration such as the confounding parameters similarly to stress. More investigations possessing different surgical or non-surgical castration techniques with larger number of goats and focusing on specific markers for stress are suggested for precise analysis.

## Methods

The present authors ensured that their manuscript reported adheres to the arrive guidelines for the reporting of animal experiments. This statement address to their manuscript that these guidelines were followed.

### Animals and housing

The study was approved by the Animal Ethics Committee of Adnan Menderes University (with no: 64583101/2015/030). A total of 16 clinically healthy, male, 12 months old and weighing 25–30 kg Alpine Goats were used in the study. The animals were obtained from the faculty farm, belonging to the Adnan Menderes University, Faculty of Veterinary Medicine. Written owner consent was available through farm manager. All goats were considered clinically healthy after a thorough clinical check together with blood and serum chemistry profile and urinalysis. The goats were given a quarantine anthelmintic drench (ricabendazole – Rizal Enjectabl, Sanovel, Istanbul, Turkey; ivermectine - Vilmectin® Enjektabl, Vilsan Veteriner Ilaclari, Ankara, Turkey) at the manufacturers recommended doses in an animal house for a 2-week period before the commencement of the study. The animals were fed twice daily at 8:00 and 16:00 with a ration of commercial goat pellets and alfa alfa hay. Water was supplied ad libitum and mineral licks were provided for free access.

### Study design, castration and treatment

Goats were enrolled, and each case received KTP (group I, *n* = 8) or control (group II, *n* = 8) in a randomized fashion, similarly to what have been described elsewhere [39]. Each group of goats was kept in suitable single boxes, which were then marked by ear tags. Goats were sedated with 0.3 mg/kg dose Xylazine-HCl intravenously [8]. Afterwards KTP (3 mg/kg) was injected via jugular vein in group I, indeed physiological saline solution (1 ml) was administered to group II. Goats were castrated by the Burdizzo (emasculatome) method. All castrations were performed by the same surgeon, who was experienced with the technique.

### Collection of blood samples and laboratory analyses

Blood samples (4 ml/sample) from all goats were collected from the jugular vein via 20-gauge 25 mm needles into 2 evacuated tubes (one that contained EDTA-K, and another that contained a coagulation activator). Blood samples were obtained 30 min before injection of Xylasine-HCl (baseline: time 0) and 6, 12, 24, 48, 72, 96 h after the end of castration.

Blood samples contained EDTA-K were used to determine hematologic variables and Fb concentration. Hematologic parameters were performed with a blood cell counter (Abacus Junior Vet 5, Diatron Messtechnik GmbH, Vienna, Austria) calibrated for goat blood; WBC and PCV counts were used for statistical analysis. Plasma Fb concentration was measured via the Millars technique [1]. Plasma Fb concentrations and hematologic variables were determined within 6 h of the same day.

Other blood samples contained a coagulation activator were used to determine other APPs. Each blood sample was centrifuged at 3000 g for 10 min and the resulting serum was transferred to plastic tubes and stored at −20 °C for analysis. All serum samples were analysed on the same day after the sample collection period.

Serum Hp and SAA concentrations were measured with a commercially available ELISA kit (Cat no: TP-801 and TP-802 for Hp and SAA, respectively, Tridelta Development Ltd., Kildare, Ireland) at the manufacturers’ recommended assay procedure. Hp and SAA concentrations were evaluated reference value versus at 630 and 450 nm, respectively, in a microplate reader (ELX-808, BioTek Instruments Inc., Vermont, USA) as mentioned in the method. Free hemoglobin possesses peroxidase activity that might be inhibited at low pH. Hp present in the blood sample reacts with hemoglobin, with a low pH demonstrates peroxidase activity by bounding to hemoglobin. SAA kit, a solid sandwich Enzyme Linked Immuno Sorbent Assay (ELİSA) performed in automated format. By the manufacturer a monoclonal antibody specific for SAA has been coated onto the wells of the microtitre strips. Obtained specimens [involving calibrators of known SAA content], were incubated into micro-wels at 37 °C together with a HRP labeled anti-SAA antibody. The presence SAA was captured between the the labeled antibody and coated microplate. The plate was washed following sampling and antibody-HRP incubation were removed within unbound material. Afterwards adding TMB, a blue product generating the colour, to those of direct proportion to the amount of SAA present in the original sample/calibrator. The reaction was finalized within the addition of stop reagent. The serum concentration of Cp was determined by measuring *p*-phenylenediamine oxidase activity as described by Ravin [37] with a spectrophotometer (UV-1601 UV-VIS Spectrophotometer, Shimadzu Corporation Tokyo, Japan).

### Statistical analysis

Statistical analysis was performed with a statistical software program (SPSS-Version 21.0, SPSS Inc., Chicago, USA). A Kolmogorov-Smirnov test was used to assess all variables for normality. For data that were not distributed normally, transformations were applied to normalize the distribution. The effects of time, group (i.e., treatment), and group-by-time interaction were assessed via and ANOVA for repeated measures. When a significant group-by-time interaction was detected, Tukey multiple comparison tests were used to compare treatments within each time period. Within each group, the baseline value was compared with the values at various time points after castration and isotonic-NaCl/ketoprofen by use of the Bonferroni correction method. Results were considered significant at values of *P* <0.05. Comparisons within and between groups were based on the final statistical model.

## Abbreviations

APPs, Acute phase proteins; APR, The acute phase response; ASG, Acid soluble glycoprotein; Cp, Ceruloplasmin; Fb, Fibrinogen; Hp, Serum haptoglobin; KTP, Ketoprofen; NSAID, Nonsteroidal anti-inflammatory drugs; SAA, Serum amyloid A
